# Study on diverse pathological characteristics of heart failure in different stages based on proteomics

**DOI:** 10.1111/jcmm.17170

**Published:** 2022-01-19

**Authors:** Jinying Liu, Hongjian Lian, Jiang Yu, Jie Wu, Xiangyang Chen, Peng wang, Lei tian, Yunfei Yang, Jiaqi Yang, Dong Li, Shuzhen Guo

**Affiliations:** ^1^ College of Traditional Chinese Medicine Chengde Medical University Chengde Hebei Province China; ^2^ School of Traditional Chinese Medicine Beijing University of Chinese Medicine Beijing China; ^3^ Alexa League Central Hospital Inner Mongolia China; ^4^ Youcare Pharmaceutical Group Drug Research Institute Beijing China; ^5^ Beijing Qinglian Biotech Co., Ltd Beijing China; ^6^ School of Basic Medical Sciences Anhui Medical University Hefei China; ^7^ State Key Laboratory of Proteomics Beijing Proteome Research Center National Center for Protein Sciences (PHOENIX Center) Beijing Institute of Lifeomics Beijing China

**Keywords:** cytoskeleton, heart failure, metabolism, proteomics

## Abstract

Heart failure is a process characterized by significant disturbance of protein turnover. To elucidate the alterations in cardiac protein expression during the various phases of heart failure and to understand the nature of the processes involved, we analysed the proteome in an established heart failure model at different time points to monitor thousands of different proteins simultaneously. Here, heart failure was induced by transverse aortic constriction (TAC) in KM mice. At 2, 4 and 12 weeks after operation, protein expression profiles were determined in sham‐operated (controls) and TAC mice, using label‐free quantitative proteomics, leading to identification and quantification of almost 4000 proteins. The results of the KEGG pathway enrichment analysis and GO function annotation revealed critical pathways associated with the transition from cardiac hypertrophy to heart failure, such as energy pathways and matrix reorganization. Our study suggests that in the pathophysiology of heart failure, alterations of protein groups related to cardiac energy substrate metabolism and cytoskeleton remodelling could play the more dominant roles for the signalling that eventually results in contractile dysfunction and heart failure.

## 1 INTRODUCTION

Heart failure (HF) is a complex process characterized by significant changes in cardiac structure, cellular function and metabolism, which contributes to the remodelling process and reduced ventricular contractility. The complex syndrome of heart failure is a major public health problem and is also a leading and increasing cause of morbidity and mortality worldwide. Despite recent advances in the treatment of HF, the 5‐year mortality rate for individuals with this disease still remains around 50% and declines slowly. The underlying molecular causes of heart failure in most heart attacks are still unknown but are anticipated to be caused by causal changes in gene and protein expression. Proteins act as molecular effectors of cells and are directly involved in all biological processes. In the context of systems biology, protein sets govern organ development and play important roles in organ functions via highly dynamic, coordinated and stage‐specific expression changes.^1^

Recently, significant improvements have been made in the analytical capabilities of biological mass spectrometry (MS)‐based proteomics, making the identification of nearly all expressed proteins in cells or tissues possible with good accuracy and reproducibility.^2^ Proteomic technology now allows us to examine global alterations in protein expression in the diseased heart and can provide new insights into cellular mechanisms involved in cardiac dysfunction. Several studies based on the proteomic analysis of HF have revealed changes of protein expression involved in cardiac energy metabolism, mitochondrial dysfunction, cardiac extracellular matrix and sarcoplasmic reticulum calcium mishandling.^3^, ^4^, ^5^, ^6^, ^7^ Finally, it is important to note that although a substantial number of processes have been recognized to play an important role in the subsequent events resulting in heart failure, it is largely unknown how the expression of proteins involved in this process is phased in time and how the expression profiles are correlated. The molecular mechanisms involved in the transition from cardiac hypertrophy to HF remain incompletely understood in particular.

This publication builds on previous research undertaken by our research team, in which heart failure was induced by transverse aortic constriction (TAC) in kunming mice.^8^ Myocardial tissue samples from different stages of the previous experiment are used to elucidate the alterations in cardiac protein expression during the various phases of heart failure and to assess the nature of the processes involved by applying proteomics technology, monitoring thousands of different proteins simultaneously at different time points. The systematic characterization of cardiac proteins across different stages of HF may provide the most valuable information and insights into our understanding of signalling pathways in pathophysiology and identification of new therapeutic targets.

## 2 MATERIAL AND METHODS

### 2.1 Animals and groups

The animals in this study all came from our team's previous research, in which heart failure was induced by transverse aortic constriction (TAC) in kunming mice. As described in the previous literature, the animals and procedures used were conducted in accordance with the ‘Guiding Principles in the Care and Use of Animals’ from the China Physiological Society and received approval from the Animal Care Committee of Beijing University of Chinese Medicine.

Briefly, mice from both sham‐operated group (*n* = 150) and transverse aortic constriction group (*n* = 200) were randomly divided into 2‐, 4‐, 6‐, 8‐ and 12‐week subgroup equally; the general condition, echocardiography and pathological features at each observation point were observed and conducted such conclusion: heart failure was successfully induced by elevated cardiac afterload caused by TAC in KM mice.^8^, ^9^ Based on the results of this study, we selected groups that can represent different stages of heart failure for in‐depth proteomics research. At 2, 4 and 12 weeks (*n* = 4) after TAC surgery, 12 mice that TAC model was only considered successful were selected randomly and were further divided into three groups: TAC 2 group, TAC 4 group and TAC 12 group. 4 mice from the sham operation group at 12 weeks were selected as the control group.

### 2.2 Echocardiography

Transthoracic echocardiography was performed on the day before the animals were sacrificed. Transthoracic echocardiography was performed for mice from TAC group and mice from sham operation group by using Vevo2100 ultrasound system (Visualsonics) and corresponding probe (MS‐400) with the centre frequency of 30 MHz and standard frame rate 449 fps. Mice were breathing anaesthetized with isoflurane and placed in the supine position after the chest hair was shaved. The chamber dimensions and left ventricular function were measured using computer algorithms as previously described.^52^ All measurements were recorded as the mean of ten consecutive cardiac cycles.

### 2.3 Myocardial tissue separation and histological analysis of the heart

After echocardiography, the animals’ chests were quickly opened, the heart was removed without the artery and the great vessels, the blood clots in the heart chambers were rinsed with ice‐cold phosphate‐buffered saline (PBS) and dried using a filter paper. The cardiac tissues were weighed and cut horizontally into two parts, and then, the upper part of tissue was fixed by paraformaldehyde, gradient dehydrated by ethanol and embedded in paraffin. Heart sections were subjected to routine haematoxylin‐eosin staining (HE staining) for microscopic observation. The left cardia tissues quick‐frozen in liquid N2 and stored at −80°C.

### 2.4 Extraction and digestion of cardiac tissue proteome

Sixteen frozen cardiac tissues samples from the TAC group at 2, 4 and12 weeks and the sham operation group at 2, 4 and12 weeks (*n* = 4) were collected for in‐depth study.

At least 1 mg samples of cardiac tissues were cut‐off and lysed in a buffer that consisted of 8 M Urea, 100 mM Tris Hydrochloride, pH 8.0, protease and phosphatase inhibitors (Thermo Fisher Scientific) for 10 min. The initial lysates were treated by ultrasonic under appropriate conditions. The lysates were centrifuged at 16,000 *g* for 10 min at 4°C, and the supernatants were retained as whole tissue extract (WTE). Bradford protein assay was used to determine the protein concentration. Approximately, 100 μg of WTE was reduced with 10 mM dithiothreitol (DTT) at 56°C and alkylated with 20 mM iodoacetamide (IAA) at room temperature in the dark. Then, WTE was digested with sequencing grade trypsin that cleaves at the C‐terminus of the Arg or Lys residues at 37°C.

### 2.5 Mass spectrometry analysis

The tryptic peptides were dried in vacuum, redissolved in 10 mM of ammonium bicarbonate buffer (pH = 10) and subjected to small scale reversed‐phase (sRP) chromatography with a self‐made C18 column. The peptides were separated into nine fractions by stepwise increasing acetonitrile (ACN) from 6 to 35% under a basic condition (pH 10).^53^ These fractions were combined into 6 samples, dried in vacuum and stored at −80°C until subsequent use for liquid chromatography tandem mass spectrometry (LC‐MS/MS) analysis. MS samples were analysed on a Q Exactive HF mass spectrometer (MS) (Thermo Fisher Scientific) interfaced with an Easy‐nLC 1000 nanoflow LC system (Thermo Fisher Scientific). In short, the samples were redissolved in 30 μl of Solvent A (0.1% formic acid in water), and one fifth of the reconstituted samples were loaded onto a self‐made reversed‐phase C18 column (2 cm × 100 μm; particle size, 3 μm; pore size, 300 Å) and then separated by a 150 μm × 12 cm silica microcolumn (homemade; particle size, 1.9 μm; pore size, 120 Å) with a linear gradient of 5–35% Mobile Phase B (0.1% formic acid in acetonitrile) at a flow rate of 600 nl/min for 75 min. By using data‐dependent strategy, MS1 in the Orbitrap was measured at a resolution of 120,000, and then, the top 20 precursors were scanned by tandem mass spectrometry with higher‐energy collision dissociation (27% of normalized collision energy and 18 s of dynamic exclusion time). Trypsin digests of 293T cells were routinely assayed as quality control samples to ensure good sensitivity and reproducibility.

### 2.6 Protein identification and quantification

Raw MS files were processed with the MaxQuant software (version 1.5.3.30), using the integrated Andromeda search engine with FDR <1% at peptide and protein level. As a forward database, National Center for Biotechnology Information (NCBI) Ref‐seq mouse proteome database (updated on 04/07/2013, 27,414 entries) was used. A reverse database for the decoy search was generated automatically in MaxQuant. Enzyme specificity was set to ‘Trypsin’, and a minimum number of seven amino acids were required for peptide identification. For label‐free protein quantification, the ‘Match Between Runs’ option was used with a matching window of 3 min to transfer MS1 identification between Runs. Default settings were used for variable and fixed modifications (variable modification: acetylation (Protein‐N terminus) and oxidation methionine (M), fixed modification: carbamidomethylation (C)). We used MaxQuant LFQ algorithm to quantitate the MS signals, and the proteins intensities were represented in iBAQ.

### 2.7 Real‐time quantitative RT‐PCR

The target‐specific primers used for real‐time quantitative reverse transcription (RT)‐PCR are shown in Table S1. Forward and reverse primers, complementary DNA template and the FastStart universal SYBR Green Master Mix (Roche) were mixed to a final volume of 20 μl. The following program was used: 95°C for 10 min, 40 cycles of 95°C for 15 s and then 60°C for 1 min. The 2−ΔΔCT method was employed to calculate the relative fold changes.

### 2.8 Statistical and bioinformatics analysis

Statistical analyses were performed with the SPSS program (SPSS version 17.0). All data were presented as the mean ± standard error of the mean (SEM). Data were analysed using a one‐way analysis of variance or a Student's *t*‐test followed by Bonferroni's method for post hoc pair‐wise multiple comparisons. The value of *p* < 0.05 was considered statistically significant.

### 2.9 Differential protein screening

Protein quantification based on the area under the curve (AUC) of precursor ions was calculated by a label‐free, intensity‐based absolute quantification (iBAQ) approach.^54^ The fraction of total (FOT) was used to represent the normalized abundance of a protein across experiments. The FOT was defined as the iBAQ of a protein divided by the total iBAQ of all identified proteins in one experiment. Missing values were replaced with zeros. iBAQ values were normalized by median centring. As the number of repeated samples was over 3, the Student's t test (with Bonferroni correction) was used to analyse the significance of differences, and more than 1.5‐fold increase or decrease protein (*p* < 0.05) was cut‐off to distinguish changes and analysis. Volcano plots and heat map were visualized using the ggplot2 packages and heatmap.2 package in R respectively.

### 2.10 Functional and pathway enrichment analysis

Gene ontology (GO) and Kyoto Encyclopedia of Genes and Genomes (KEGG) pathway function enrichment analysis were performed based on DAVID online tool, an online platform for the high‐throughput functional annotation bioinformatics.^55^ The GO enrichment analysis, including the cellular component (CC), the biological process (BP) and the molecular function (MF) terms, was regarded as significant with *p* < 0.05. In the KEGG pathway enrichment analysis, enriched pathways were regarded as significant with *p* < 0.05.

### 2.11 PPI network construction and analysis of modules

The Search Tool for the Retrieval of Interacting Genes/Proteins (STRING) is mainly used to predict and construct protein‐protein interactions. In this study, STRING database was used to construct the PPI interactions of the 101 overlapping differentially expressed proteins protein‐protein, and combined score ≥0.4 was identified as a major selection condition.^56^ In addition, the PPI network was visualized by Cytoscape software.^57^ The Molecular Complex Detection (MCODE) plug‐in by Cytoscape was used to filter the modules in PPI network, with the MCODE scores >4 and the number of nodes >5 as the cut‐off criterion.^58^

## 3 RESULTS

### 3.1 Pressure overload causes compensated hypertrophy followed by heart failure with normal and then impaired EF

In this paper, we studied four groups of mice at three different time points. The TAC groups were sacrificed at 2, 4 and 12 weeks after inducing left ventricular (LV) pressure overload by TAC and the sham‐operated mice at 12 weeks after surgery. We balanced the number of TAC and sham mice, with four samples analysed at each time point.

**FIGURE 1:**
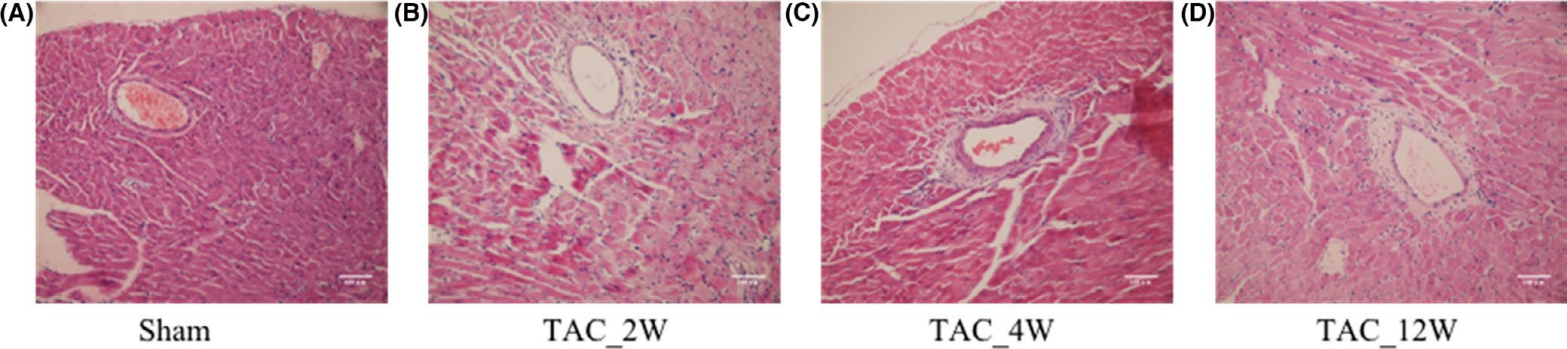
Haematoxylin and eosin staining (HE) of the mouse heart from various groups at the two indicated magnifications^9^

Transthoracic echocardiography was performed on the day before the animals were sacrificed. Echocardiographic and anatomical data from all mice have been previously published.^8^ The major features of the surgical model used in the present study are summarized in the Table and in Figure 1. Table 1 showed basic morphometric parameters of animals analysed 2, 4 and 12 weeks after the TAC operation. In short, heart weights and heart weight to body weight ratios in all the TAC groups were higher than in the age‐matched sham‐operated groups, indicating a significant degree of hypertrophy in all TAC groups.^7^ The segmental decrease in left ventricular ejection fraction was paralleled by an increase in left ventricular end‐diastolic volume and end‐systolic volume reflecting the time course from compensated left ventricular hypertrophy to development of LV systolic dysfunction during the second, fourth and the twelfth week.

**TABLE 1:** Echocardiographic parameters of hearts subjected to pressure overload

Group	*N*	LVAWd (mm)	LVAWs (mm)	LVIDd (mm)	LVIDs (mm)	LVPWd (mm)	LVPWs (mm)	EF (%)	FS (%)	LVEDV (µL)	LVESV (µL)	LVM (mg)	LVMI (mg/g)
Sham	4	1.52 ± 0.11	2.13 ± 0.12^#^	4.686 ± 0.13**	3.09 ± 0.18**	1.01 ± 0.09**	1.39 ± 0.08**	63.00 ± 3.15	34.22 ± 2.25	101.99 ± 6.25**	38.29 ± 5.33	287.75 ± 23.82	4.50 ± 0.29
TAC_2W	4	1.25 ± 0.06	1.90 ± 0.07	3.75 ± 0.12*	2.14 ± 0.21*	1.49 ± 0.13*	1.95 ± 0.20*	74.52 ± 4.61^#^	43.28 ± 4.06^#^	60.26 ± 4.68*	15.87 ± 3.77	233.59 ± 20.20	6.03 ± 0.40*
TAC_4W	4	1.31 ± 0.03	1.73 ± 0.05*	4.20 ± 0.39	3.05 ± 0.53	1.27 ± 0.10	1.71 ± 0.06	54.61 ± 9.78**	29.31 ± 6.90**	81.54 ± 16.61	41.67 ± 14.24	258.59 ± 44.96	4.83 ± 0.62
TAC_12W	4	1.31 ± 0.03	1.80 ± 0.02*	5.41 ± 0.61* ^,^ ** ^,^ ^#^	4.45 ± 0.72* ^,^ ** ^,^ ^#^	1.21 ± 0.05	1.40 ± 0.09**	36.37 ± 3.21* ^,^ ** ^,^ ^#^	17.72 ± 1.77* ^,^ **	143.03 ± 12.91* ^,^ ** ^,^ ^#^	90.31 ± 6.41* ^,^ ** ^,^ ^#^	361.59 ± 24.57	5.88 ± 0.34* ^,^ ^#^

Note

Data are mean ± SD or Percentage (%). Sham, the group of sham‐operated mice; TAC_2w, the group of 2 weeks after TAC operation; TAC_4w, the group of 4 weeks after TAC operation; TAC_12w, the group of 12 weeks after TAC operation.

Abbreviations: EF, left ventricular ejection fraction; FS, left ventricular midwall fractional shortening; LVAWd, left ventricular end‐diastolic anterior wall thickness; LVAWs, left ventricular end‐systole anterior wall thickness; LVEDV, left ventricular end‐diastolic volume; LVESV, left ventricular end‐systolic volume; LVIDd, left ventricular internal diastolic diameter; LVIDs, left ventricular internal systolic diameter; LVM, calculated left ventricle mass; LVMI, left ventricle mass index; LVPWd, left ventricular posterior wall thickness in diastole; LVPWs, left ventricular posterior wall thickness in systole.

* *p* < 0.05, compared with the sham operation group.

** *p* < 0.05, compared with the group of 2 weeks after TAC operation.

# *p* < 0.05, compared with the group of 4 weeks after TAC operation.

The heart sections were stained with haematoxylin and eosin staining (HE) to analyse the degree of cardiac pathological remodelling. Figure 1 shows the results of HE stains. The myocardial cells of the Sham group (Figure 1A) were presented in an ordered arrangement, and only a small amount of collagen was distributed in the myocardial interstitial. The TAC group at 2 weeks showed that the normal structure of the myofibrils was disappeared, the vessel lumen was larger, the vessel wall was thicker, and the connective tissue around the vessel wall proliferated nearby. (Figure 1B). At 4 and 12 weeks of the TAC groups, the above‐mentioned pathological changes were aggravated, accompanied by hypertrophy of cardiac myocytes, broadening of myocardial fibre bundles and significantly increase of fibrosis progressively (Figure 1C,D).

In conclusion, these results suggest that our model of pressure overload leads to compensatory hypertrophy (at 2th week) followed by heart failure, whereas systolic function was maintained (at 4th week), and then heart failure with impaired ejection fraction (at 12th week).

### 3.2 Proteomic profiles differ between the TAC groups and the sham operation group

**FIGURE 2:**
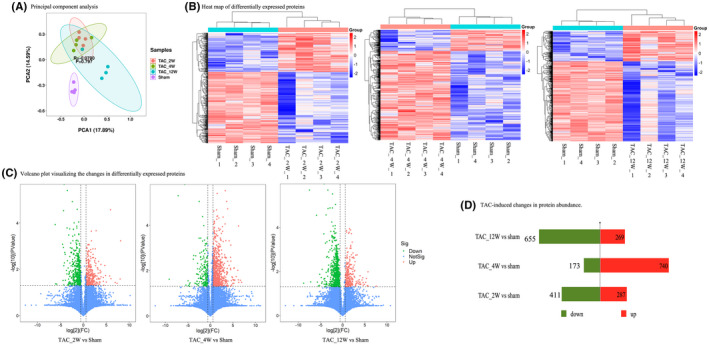
Proteomics of the TAC groups compared to the sham operation group. (A), Principal component analysis (PCA) of the sham operation group and the TAC groups (2‐week, 4‐week and 12‐week) showing a clear difference in proteomic profiles. (B), Heat map of differentially expressed proteins showing significant differences in abundance between the sham operation group and the TAC group at 2, 4 and 12 weeks. Each row in the heat map corresponds to data from a single protein, whereas columns correspond to individual samples. The branching dendrogram corresponds to the relationships among samples, as determined by clustering using identified differentially expressed proteins. Increases and decreases in protein abundance are shown on a continuum from red to blue respectively. (C), Volcano plot visualizing the changes of differentially expressed proteins between the sham operation group and the TAC groups at 2, 4 and 12 weeks. Proteins whose expression was significantly increased in the TAC groups are in the upper right section, and proteins whose expression was significantly decreased are in the upper left section. The x‐axis represents log2 fold change in the TAC groups (2‐, 4‐ and 12‐week) compared with the sham operation group. (D), Number of proteins with at least 1.5‐fold change (with *p* < 0.05) in abundance the TAC groups vs. the sham group were shown (red: upregulated proteins; green: downregulated proteins)

Principal component analysis (Figure 2A) showed that the sham group was different from the TAC groups, and also indicated considerable overlap between the TAC groups. We focused on the identification of up‐ and down‐regulation of spot intensities where the fold change was at least 1.5 (with *p* < 0.05). Hierarchical clustering of differentially expressed proteins readily separated the TAC groups and the sham operation group (Figure 2B). Compared with the sham operation group, the TAC group at 2 weeks was identified with 689 differentially expressed proteins, with 287 upregulated and 411 downregulated. At 4 weeks, 913 differentially expressed proteins were identified in the TAC group, of which 740 were higher than those in the sham group and 173 were lower than those in the sham group. At 12 weeks, 942 differentially expressed proteins were identified in the TAC group, among which 269 were more expressed than those in the sham group and 655 were less expressed than those in the sham group (Figure 2C,D).

### 3.3 Functional categorization of differentially expressed proteins

**FIGURE 3:**
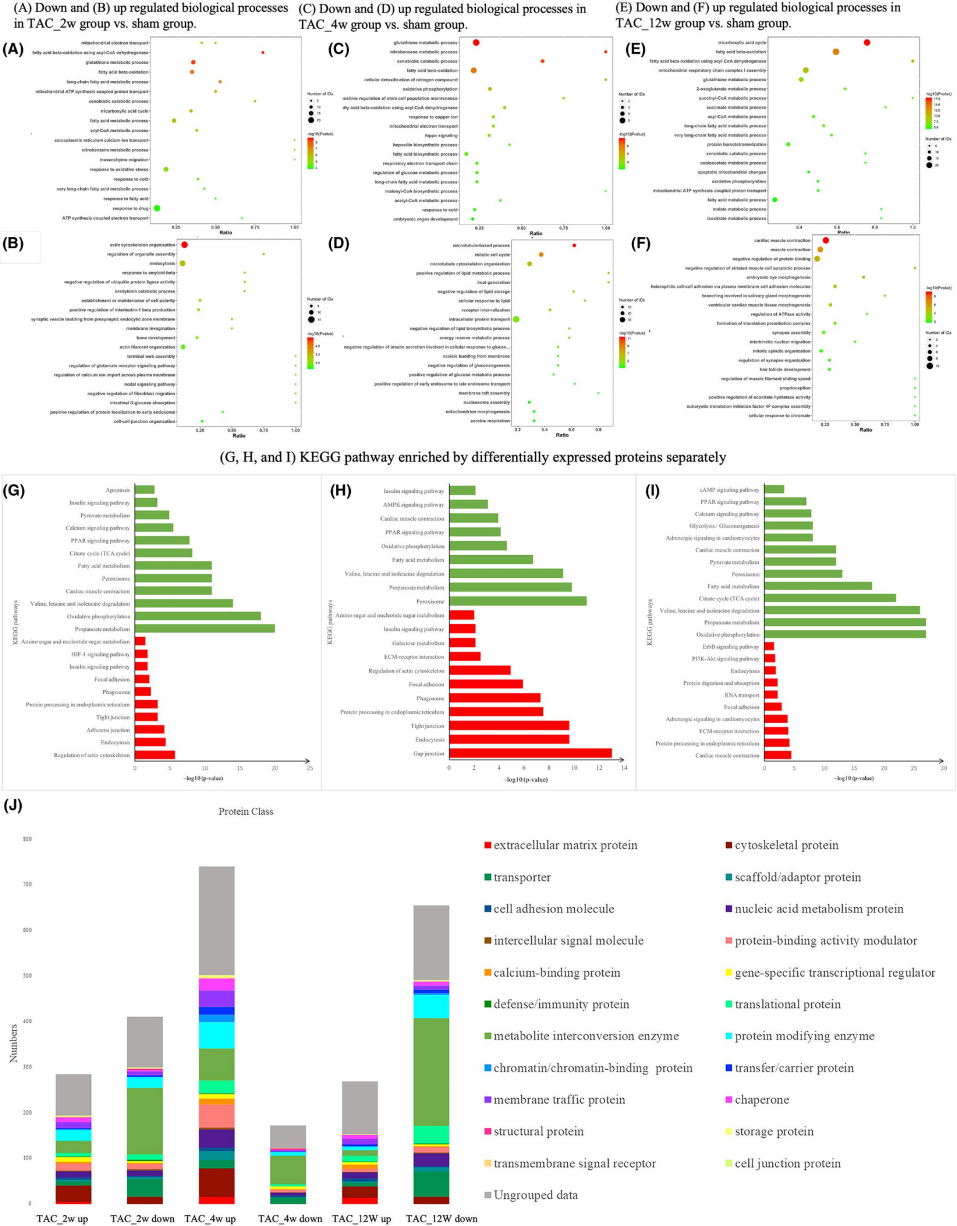
Gene ontology and KEGG annotation for the up‐ and downregulated proteins separately. (A) Down‐ and (B) Upregulated biological processes in TAC_2w group vs. sham group. (C) Down‐ and (D) Upregulated biological processes in TAC_4w group vs. sham group. (E) Down‐ and (F) Upregulated biological processes in TAC_12w group vs. sham group. (G, H, and I) KEGG pathway enriched by differentially expressed proteins separately (Only representative items were listed); red represented upregulated proteins, and green represented downregulated proteins; The rectangle represents−log10 (p‐value). (G) Up‐ and downregulated pathways in TAC_2w group vs. sham group. (H) Up‐ and downregulated pathways in TAC_4w group vs. sham group. (I) Up‐ and downregulated pathways in TAC_12w group vs. sham group. (J), ‘Protein class’ classification from the PANTHER database (http://pantherdb.org/). The bar chart represented the classification of the differentially expressed proteins according to the GO categories. The y‐axis showed the number of proteins against a total number of differentially expressed proteins

To investigate representative proteins in each phase and understand their biological significance, we performed gene ontology (GO) and Kyoto Encyclopedia of Genes and Genomes (KEGG) pathway analysis on differentially expressed proteins in the three phases (Figure 3). At 2 weeks, down‐regulation of proteins were involved in propionate metabolism, oxidative phosphorylation, valine, leucine and isoleucine degradation, cardiac muscle contraction, peroxisome, fatty acid metabolism, citrate cycle (TCA cycle), peroxisome proliferator‐activated receptor (PPAR) signalling pathway, calcium signalling pathway, pyruvate metabolism, insulin signalling pathway and apoptosis. Up‐regulation proteins mainly involved in processes regulating actin cytoskeleton, endocytosis, adherens junction, tight junction, protein processing in endoplasmic reticulum, phagosome, focal adhesion, insulin signalling pathway, HIF‐1 signalling pathway, and amino sugar and nucleotide sugar metabolism. At 4 weeks, gap junction, endocytosis, tight junction, protein processing in endoplasmic reticulum, phagosome, focal adhesion, regulation of actin cytoskeleton, extracellular matrix (ECM)‐receptor interaction, galactose metabolism, insulin signalling pathway, amino sugar and nucleotide sugar metabolism were greatly upregulated, while the downregulated proteins mainly modulated apoptosis, calcium signalling pathway and metabolic pathway including propanoate metabolism, oxidative phosphorylation, fatty acid metabolism, TCA cycle and pyruvate metabolism. At 12 weeks, the upregulated proteins mainly modulated cardiac muscle contraction, protein processing in endoplasmic reticulum, ECM‐receptor interaction and adrenergic signalling in cardiomyocytes, while the downregulated proteins mainly modulated oxidative phosphorylation, TCA cycle, glycolysis/gluconeogenesis and cyclic adenosine monophosphate (cAMP) signalling pathway. In short, among the most significantly downregulated processes, energy pathways, including oxidative phosphorylation, fatty acid metabolism, the TCA cycle and amino acid metabolism were dominated in the three TAC groups. Upregulated processes were cytoskeleton organization, cell‐matrix adhesion and response to endoplasmic reticulum stress.

Next, in order to link the biological function with the identified proteins, we classified the differentially expressed proteins according to GO categories (Figure 3J). As a result, we found a large proportion of extracellular matrix proteins and cytoskeletal proteins in the upregulated proteins in each TAC group. Interestingly, we also found an enrichment of metabolite interconversion enzymes in the downregulated proteins in each TAC group. It should be noted that although the three groups of TAC shared great similarities in the biological process modulation and KEGG pathway analysis, the biological events that differentially expressed proteins modulated were different.

Further, we searched for genetic mutations in these differentially expressed proteins, which had been previously shown to be associated with risk of cardiovascular disease in other studies (Table S2).

### 3.4 Functional annotation of overlapping differentially expressed proteins in all three TAC groups

**FIGURE 4:**
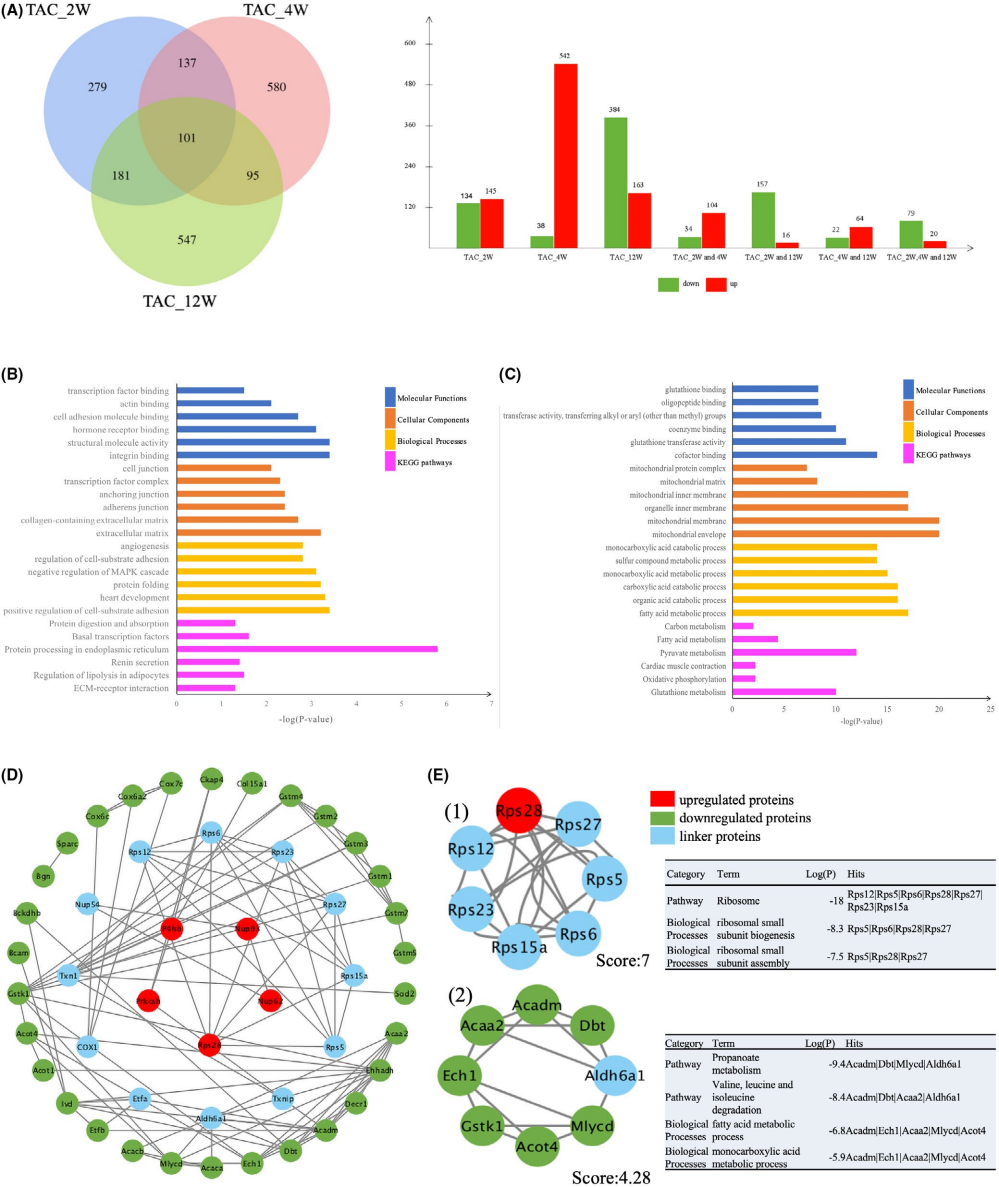
Bioinformatics analysis for the differentially expressed proteins in three TAC groups. (A), Identification of overlapping differentially expressed proteins in three TAC groups through Venn diagrams (http://bioinformatics.psb.ugent.be/webtools/Venn/). Each coloured ellipse in the Venn diagram represents a Group Set, and the number stands for the number of differentially expressed proteins in each cross area. The bar chart represented the number of up‐ and downregulated proteins in each cross area according to the Venn diagram (red: upregulated proteins; green: downregulated proteins). A total of 101 overlapping proteins were obtained from three time points after TAC, with 79 downregulated proteins and 20 upregulated proteins. (B) Upregulated and (C) downregulated GO and KEGG pathway enrichment analysis of overlapping proteins (6 representative items were listed). Four different colours separately represent four different categories: molecular function, cellular component, biological process and KEGG pathway. (D), Protein‐protein interaction network of the 101 overlapping proteins with a score >0.7. Disconnected nodes were hiding in the network. Each node stands for a gene or a protein, and edges represent the interactions between the nodes. Red nodes represent upregulated proteins; green nodes stand for downregulated proteins; and blue nodes stand for the linker proteins. (E), Two significant modules in PPI network and GO and KEGG analysis of significant modules: (1) Module 1 and (2) module 2

We further used Venn diagram to analyse overlapping proteins of all the differentially expressed proteins among these three TAC groups (Figure 4A). Among them, 101 overlapping proteins were obtained from the three groups, including 79 upregulated proteins and 20 downregulated proteins. Based on those proteins, significant results of the GO and KEGG pathway enrichment analyses were shown in Figure 4B,C respectively. The upregulated proteins were mainly enriched in the integrin binding, structural molecule activity, hormone receptor binding, cell adhesion molecule binding and transcription factor binding, whereas downregulated proteins were mainly enriched in the cofactor binding, glutathione transferase activity, coenzyme binding, transferase activity, transferring alkyl or aryl (other than methyl) groups, oligopeptide binding and glutathione binding. Upregulated proteins related to cellular component were mainly enriched in the extracellular matrix, collagen‐containing extracellular matrix, adherents’ junctions, anchoring junction, transcription factor complex and cell junction, while downregulated proteins were mainly enriched in mitochondrial envelope, mitochondrial membrane, organelle inner membrane, mitochondrial inner membrane, mitochondrial matrix and mitochondrial protein complex. Upregulated proteins modulating biological processes were mainly enriched in the positive regulation of cell‐substrate adhesion, heart development, protein folding, negative regulation of mitogen‐activated protein kinase (MAPK) cascade, regulation of cell‐substrate adhesion and angiogenesis, whereas downregulated proteins were mainly enriched in fatty acid metabolic, organic acid catabolic, carboxylic acid catabolic, monocarboxylic acid metabolic, sulphur compound metabolic and monocarboxylic acid catabolic. The KEGG pathway results revealed that the upregulated proteins are significantly enriched in ECM‐receptor interaction, regulation of lipolysis in adipocytes, renin secretion, protein processing in endoplasmic reticulum, basal transcription factors and protein digestion, whereas downregulated proteins were closely associated with glutathione metabolism, oxidative phosphorylation, pyruvate metabolism fatty acid metabolism, carbon metabolism and cardiac muscle contraction.

Considering that proteins rarely work alone, it was necessary to study the interactions among proteins. Based on the STRING database, the PPI network was established by retrieving the 101 overlapping differentially expressed proteins. After removing the disconnected nodes in Cytoscape software, the results showed that 47 nodes and 101 edges were established in this network with score >0.7 (Figure 4D). Moreover, 2 representative network modules that met the criteria of MCODE score >4 and the number of nodes >5 were extracted from PPI network. In addition, the results of GO and KEGG pathway analysis for each module showed that these proteins included in these modules were highly involved in fatty acid metabolic pathway (Figure 4E).

### 3.5 Changes in the expression of selected genes by RT‐PCR

**FIGURE 5:**
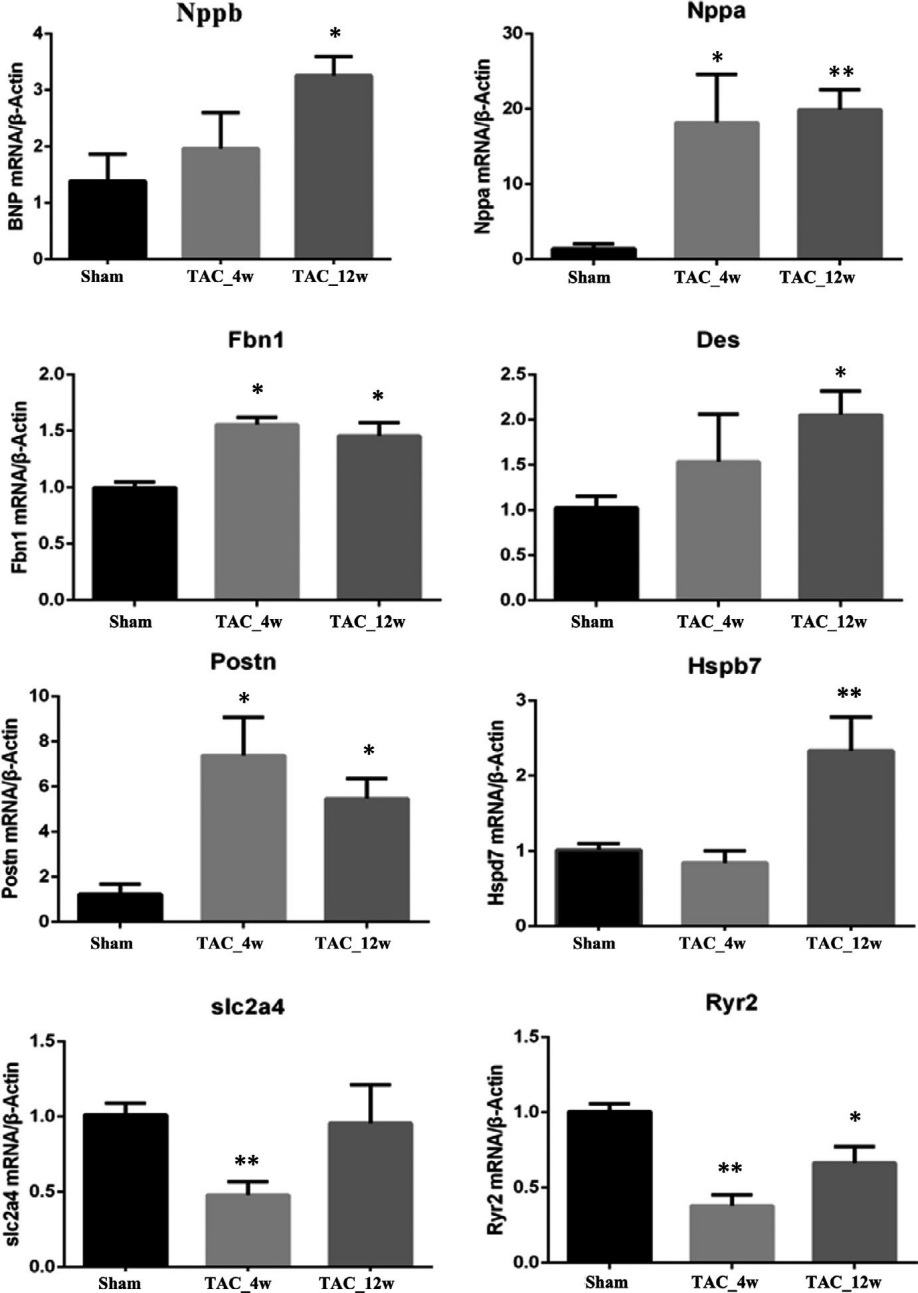
Expression of selected genes by RT‐PCR. *p < 0.05, compared with the sham operation group. **p < 0.01, compared with the sham operation group

To validate our proteomic results, reverse transcription‐polymerase chain reaction (RT‐PCR) was employed to validate 8 genes encoding 8 representative proteins listed in Table S1. As shown in Figure 5, there was a strong correlation between expression values investigated by RT‐PCR analysis and those measured by proteomic analysis. At 4 weeks after TAC operation, five of these 8 genes were found significantly changed with three significant upward trends and two significant downward trends, while at 12 weeks after TAC operation, seven of these 8 genes were found significantly changed with six significant upward trends and one significant downward trend. However, RYR2 expression was found significantly changed in both time points when measured by RT‐PCR, which was not significantly different in proteomic analysis. In addition, in proteomics, DES and POSTN increased only at 4 weeks, while HSPB7 increased at both 4 and 12 weeks.

## 4 DISCUSSION

In the present study, heart failure model in mice by TAC went through three stages: compensatory hypertrophy, heart failure with normal ejection fraction and diastolic dysfunction, and heart failure with decreased ejection fraction, which is consistent with other studies.^10^ This study outlines an overview of differences in the protein profiles during the different phases of transition to heart failure, identifying distinct protein expression patterns in evolving heart failure.

### 4.1 Disturbed energy metabolism occurs prior to the onset of heart failure

Metabolic regulation is inextricably linked with cardiac function. Metabolic remodelling in HF is characterized by a reduced energy production and alterations in the source of energy substrates.^11^, ^12^ At all three different time points of the TAC groups, the most striking change observed in proteins expression is energy metabolism. Proteins involved in oxidative phosphorylation and fatty acid metabolism are in general downregulated very early, and this change persists from the stages of compensatory cardiac hypertrophy to the stages of heart failure with impaired ejection fraction. Interestingly, there was a coordinately reduced expression of proteins modulating different steps of fatty acid metabolism, such as fatty acid‐binding proteins (transport of fatty acids in cytoplasm), long‐chain fatty acid—CoA ligase (catalyses the conversion of long‐chain fatty acids to their active form), carnitine O‐palmitoyltransferase 2 (transport of long‐chain fatty acids from cytoplasm to mitochondrial matrix), enoyl‐CoA hydratase and acyl‐CoA dehydrogenases (mitochondrial β‐oxidation) and peroxisomal acyl‐coenzyme A oxidase (peroxisomal fatty acid oxidation). This change is accompanied by the gradually increasing of upregulated proteins linked to glucose metabolism in the stage of cardiac hypertrophy and heart failure with preserved ejection fraction; however, in the stage of heart failure with reduced ejection fraction glycolysis related proteins is decreased. This result is consistent with previous reports that cardiac hypertrophy and heart failure due to imposed pressure overload are characterized by a decrease of fatty acid metabolism oxidation rates and fatty acid‐related proteins.^12^, ^13^, ^14^

Moreover, the heart has been shown to undergo a shift in energy substrate preference from fatty acid to glucose during cardiac hypertrophy and heart failure in most animal studies.^15^, ^16^ This energy metabolic switch is associated with reductions in fatty acid oxidation and a concurrent increase in glucose utilization, which has been interpreted as reactivation of ‘foetal’ gene expression.^17^ The increase in glucose utilization is associated with overexpression of glucose transporter 1 and decreased expression of glucose transporter 4.^13^, ^18^, ^19^ According to Razeghi research, reactivation of ‘foetal’ metabolic gene profile in the failing adult heart occurs by the downregulating adult gene transcripts (eg GLUT4) rather than by the upregulating foetal genes (eg GLUT1).^20^ In our study, the down‐regulation of glucose transporter 4 and the upregulation of glucose metabolism‐regulating proteins were observed in mice at 2 and 4 weeks after TAC, which could contribute to increase glucose intake. Since ATP generation from glucose metabolism can increase oxygen efficiency compared to fatty acid metabolism, this metabolic remodelling at the initial stages of HF upon pressure overload may be an adaptive mechanism to cope with the extra workload.^21^ This change may explain the compensatory hypertrophy with elevated cardiac function at 2 weeks and heart failure with cardiac diastolic dysfunction at 4 weeks, whereas systolic function was maintained. In agreement with this notion, other studies in mice with transverse aortic constriction‐induced pressure overload have also demonstrated that mice with TAC operation exhibited an increase in glucose utilization as early as 1 day after TAC and that they were further increased over time.^22^, ^23^ Considering that ATP generated from glycolysis alone contributes less than 5% of the total ATP consumed in heart, the amount of ATP produced by upregulating glycolysis metabolism is far from being sufficient to meet the increase of metabolic demand and wall stress.^24^ Moreover, impaired fatty acid metabolism and beta‐oxidation can lead to the excess deposition of toxic lipid products that adversely affect cardiomyocyte function.^25^ It has been proposed that if the reduction of fatty acid metabolism cannot be fully compensated by the increased glucose oxidation, it may lead to the limitation of substrate utilization and mitochondrial dysfunction, eventually leading to energy starvation and accelerating the decline of cardiac function.^26^ Therefore, we hypothesize that return to ‘foetal’ metabolic gene profile seems to be critical for short‐term adaptation to pressure overload, which may prove to be maladaptive in the long run. As we expected, the state of severe heart failure—HF with impaired ejection fraction (at 12 weeks) can be characterized by a significant decrease in energy metabolism pathways and protein expression, including fatty acid oxidation, tricarboxylic acid cycle and glycolytic pathway. Our finding is consistent with the results that metabolic processes, including glycolysis, are decreased in severe heart failure.^20^, ^27^ Of course, our finding seems to be discordant with previous reports that rate of glucose uptake was increased in the hypertrophied rat heart.^28^, ^29^ Apparent discrepancies in cardiac fatty acid oxidation, glycolysis and glucose oxidation between the studies can attributed to either the severity of failure or different animal models resulting in different degrees of afterload that characterizes the transition to heart failure. Our observation emphasizes the importance of flexibility of substrate utilization for normal heart function. The heart must adjust its preference for substrate utilization to accommodate a perturbation in energy demand; otherwise, this flexibility may deteriorate with the change of pathological conditions.

### 4.2 Impairment of cytoskeleton and extracellular matrix during progression of cardiac hypertrophy to heart failure

Heart failure is characterized by global myocardial remodelling, mainly consisting of myocardial fibrosis associated with changes in the cytoskeletal in cardiomyocytes, which represent structural correlates of reduced ventricular function.^30^ Changes of the cytoskeleton and extracellular matrix may contribute to myocardial remodelling.^31^ The cytoskeleton system of cardiomyocytes plays a key role in maintaining cell morphology, and the extracellular matrix (ECM) controls cytoskeletal mechanics and structure. Therefore, both the cytoskeleton and ECM are essential for mediating structural remodelling and functional responses within the myocyte.^32^

The excessive numbers of cytoskeletal proteins and extracellular matrix proteins, particularly actin cytoskeletal proteins, microtubules and integrins, were detected as early as 2 weeks, which is in line with the increased left ventricular mass 2 weeks after TAC. Integrins transmitting mechanical stress to the cytoskeleton is thought to represent a proximal step of intracellular mechanical signalling that leads to the rearrangement of the overall cytoskeleton.^33^ Cytoskeleton reorganization is important in maintaining cell homeostasis. Generally speaking, this phase gives a clue that an adaptive response of the cardiomyocyte cytoskeleton underwent global rearrangement to cope with increased workload, since the stress compensation by increased wall thickness requires an adaptation in cell structure.^34^, ^35^ In the period of impaired diastolic pressure but stable systolic heart function, the increased amount of cytoskeleton displays a significant and constant alteration of the actin‐cytoskeleton signalling. Increase in cytoskeletal proteins plays a compensatory role in maintaining ventricular wall tension during compensatory hypertrophy, but may worsen ventricular diastolic dysfunction by decreasing ventricular compliance and further increase the contractile load of marginalized cardiomyocytes, and thus damage cardiac function.^36^, ^37^ Desmin as a major intermediate filament protein supports the sarcomere, whose increase represents a novel factor contributing or paralleling the development of diastolic dysfunction.^36^A stable dense microtubule network decorated by microtubule‐associated protein 4 is a maladaptive persistent characteristic of cardiac hypertrophy caused by pressure overload that leads to heart failure by interfering with cardiac contraction and intracellular transport.^38^ The results found in our study that upregulation of desmin during the stage of cardiac hypertrophy and HF with impaired diastolic pressure, and upregulation of microtubule‐associated protein 4 in the period of HF with decreased ejection fraction can also prove these conclusions. It has been proposed that an increase in cytoskeletal stiffness and apparent viscosity could establish a causal relationship between the cytoskeleton and extracellular matrix in cardiac hypertrophy and failure.^39^, ^40^ Not surprisingly, in the end stage of heart failure, more extracellular matrix proteins (eg secreted protein acidic and rich in cysteine; integrin alpha‐5; collagen alpha‐1 (XV) chain;fibrillin‐1; fibulin‐2; galectin‐1; tenascin XB; and decorin) were observed, accompanied by the decrease of muscle contraction proteins and ion channel‐related proteins (eg caveolin‐3; desmoplakin; myosin‐binding protein C, cardiac‐type; myomesin 2; voltage‐dependent anion‐selective channel protein 1; potassium voltage‐gated channel subfamily KQT member 1; dynamin‐like 120 kDa protein, mitochondrial; ryanodine receptor 2; and junctophilin‐2). Subsequently, we focus on a few selected altered proteins whose potential importance is supported by the publications. In pressure overloaded hearts, matricellular proteins, such as secreted protein acidic and enrichment in cysteine and tenascin XB, may act by stimulating a fibrogenic program in cardiac fibroblasts.^41^ Extensive evidence suggests that the changes in the myocardial ECM network in response to pressure overload not only trigger fibrosis and perturbs cardiomyocyte relaxation, increasing myocardial stiffness and causing diastolic dysfunction, but also result in dilative remodelling and systolic dysfunction. Although the basis of the transition may involve several different cellular processes, the myocardial ECM network is not only essential to regulate the geometry and function of the heart, but also contributes to cardiac homeostasis.^42^ Desmoplakin interconnects the desmosomal junction to the cytoskeletal‐based intermediate filament system, which is critical for maintaining cardiac desmosomal cell‐cell adhesion integrity and function.^43^, ^44^ Cardiac myosin‐binding protein C, a component of the thick filament of heart muscle, is functionally related to cardiac diastolic function and hypertrophy.^45^ Ryanodine receptor 2 and junctophilin‐2 are proteins related to changes in cardiac contraction in response to cellular calcium ion homeostasis.^46^, ^47^ Ryanodine receptor 2 constituting the major intracellular Ca^2+^ release channel in the cardiac sarcoplasmic reticulum has been linked to cardiac arrhythmias and heart failure.^48^, ^49^ Mutations in the cardiac ryanodine receptor (RYR2) gene have been reported to cause arrhythmogenic right ventricular cardiomyopathy.^50^ Increasing evidence suggests down‐regulation of junctophilin‐2 contributes to T‐tubule remodelling, impaired cardiac contractility, loss of excitation‐contraction coupling and heart failure progression.^51^, ^52^ In closing, we conduct that cytoskeleton and ECM seem to play a critical role in the maintenance of structural and mechanical integrity of the contractile apparatus in muscle tissues.

## 5 CONCLUSIONS

The mechanisms underlying heart failure are diverse and complex. In this study, we provide a general picture of alterations of protein profiles. Our study focused on the proteins involved in energy and cytoskeleton as well as extracellular matrix protein during the different stages of transition from cardiac hypertrophy to heart failure. In conclusion, these pathophysiological processes occurred at different stages of HF, contribute to or worsen the progression to overt systolic HF phenotype. Our study suggests that a better understanding of the complex phenotypic response in HF and its associated protein expression changes will help to better understand the pathological changes of the disease, and it is also a prerequisite for formulating precise target treatment strategies.

## 6 ETHICS STATEMENT

The animals and procedures used were conducted in accordance with the ‘Guiding Principles in the Care and Use of Animals’ from the China Physiological Society and received approval from the Animal Care Committee of Beijing University of Chinese Medicine. Extensive efforts were made to ensure minimal suffering of the included animals.

## CONFLICTS OF INTEREST

The authors report no conflicts of interest in this work.

## AUTHOR CONTRIBUTIONS

**Jinying Liu:** Data curation (equal); Formal analysis (equal); Methodology (equal); Resources (equal); Writing – original draft (equal); Writing – review & editing (equal). **Hongjian Lian:** Data curation (equal); Formal analysis (equal); Methodology (equal); Resources (equal); Validation (equal); Writing – original draft (equal); Writing – review & editing (equal). **Jiang Yu:** Data curation (equal); Methodology (equal); Validation (equal); Writing – original draft (equal); Writing – review & editing (equal). **Xiangyang Chen:** Methodology (equal); Software (equal); Validation (supporting); Writing – original draft (supporting); Writing – review & editing (supporting). **Peng Wang:** Conceptualization (supporting); Writing – original draft (supporting). **Lei Tian:** Writing – original draft (supporting). **Yunfei Yang:** Writing – original draft (supporting). **Shuzhen Guo:** Funding acquisition (lead); Project administration (lead); Resources (lead); Supervision (lead); Writing – review & editing (equal). **Dong Li:** Funding acquisition (equal); Project administration (equal); Resources (equal); Software (equal); Supervision (lead); Writing – review & editing (equal). **Jie Wu:** Methodology (equal); Software (equal); Validation (equal); Writing – original draft (equal); Writing – review & editing (equal). **Jiaqi Yang:** Methodology (equal); Software (equal); Validation (equal); Writing – original draft (equal); Writing – review & editing (equal).

## Supporting information

Fig S1

Fig S2A

Fig S2B

Fig S3A

Fig S3G

Fig S3J

Fig S4A

Supplementary Material

Supplementary Material

Table S1

Table S2

## Data Availability

The datasets used and analysed during the current study are available from the corresponding author on reasonable request.
