# Supplementary figures and images for: Study on diverse pathological characteristics of heart failure in different stages based on proteomics
Source: J Cell Mol Med. 2022 Jan 19;26(4):1169–82. doi: 10.1111/jcmm.17170 (PMC8831959; doi:10.1111/jcmm.17170)

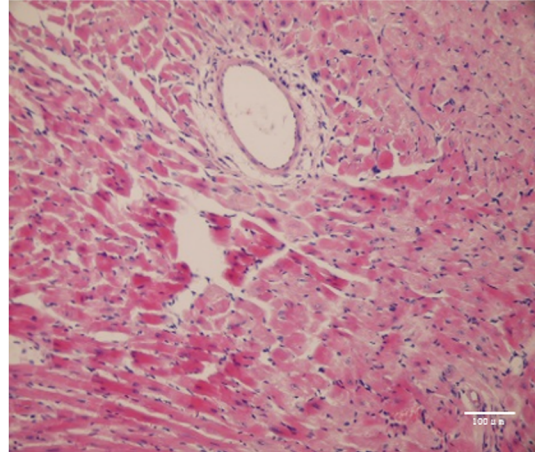

TAC-2w

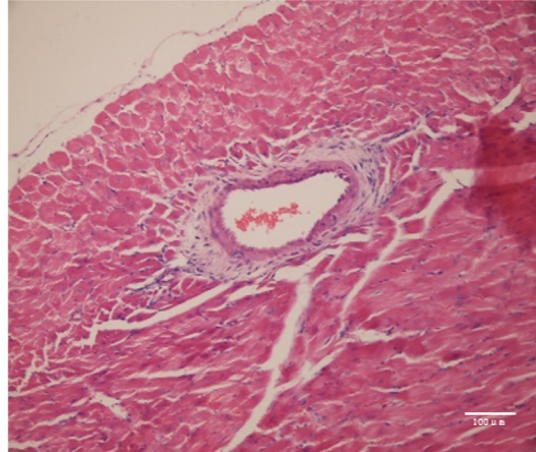

TAC-4w

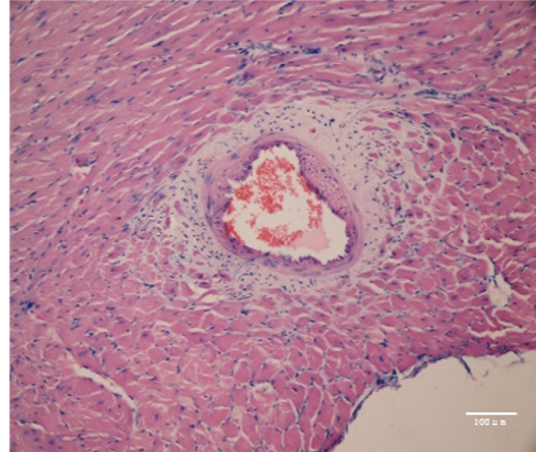

TAC-6w

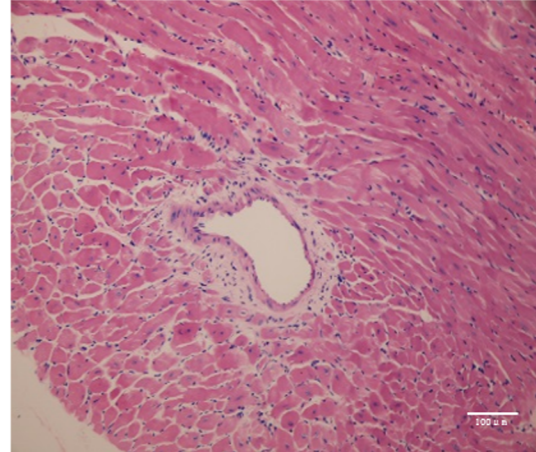

TAC-8w

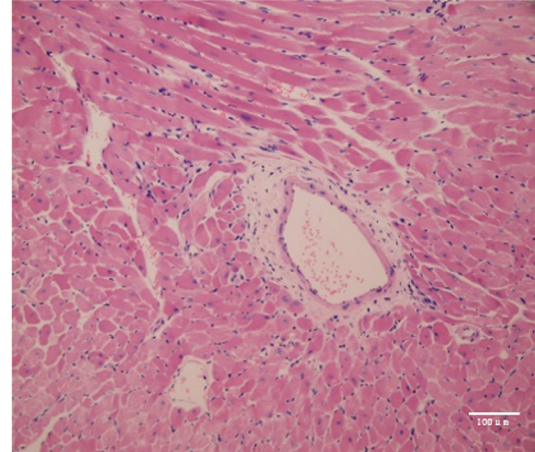

TAC-12w

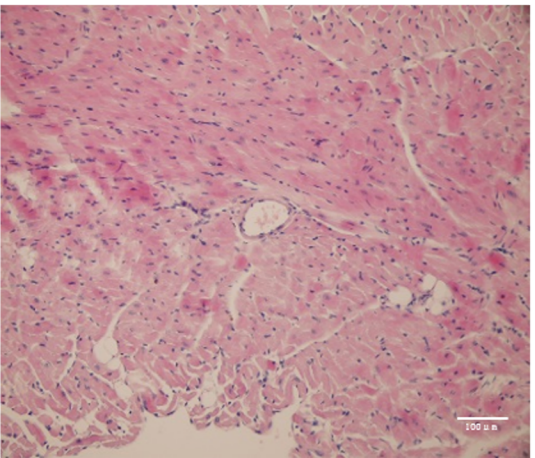

Sham-2w

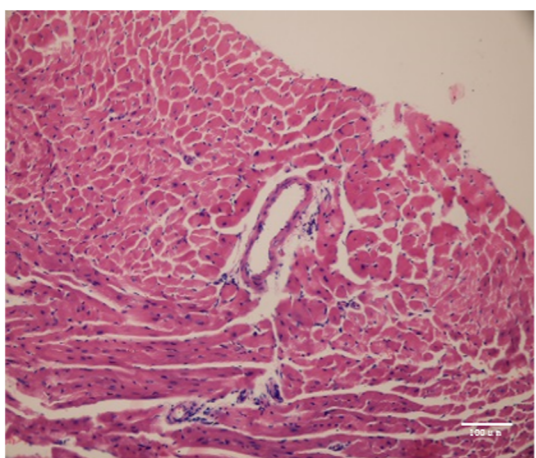

Sham-4w

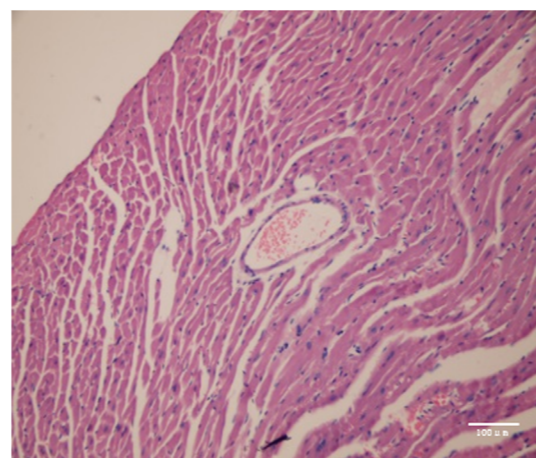

Sham-6w

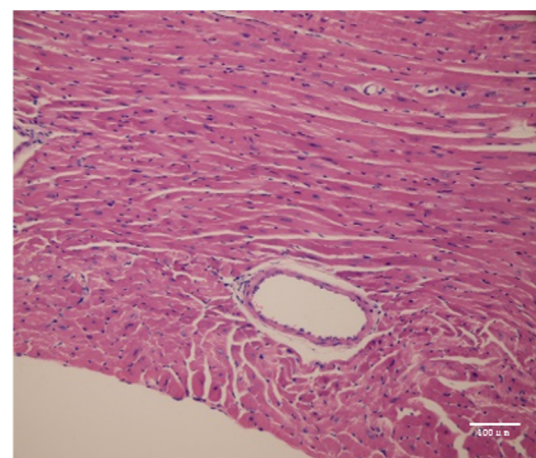

Sham-8w

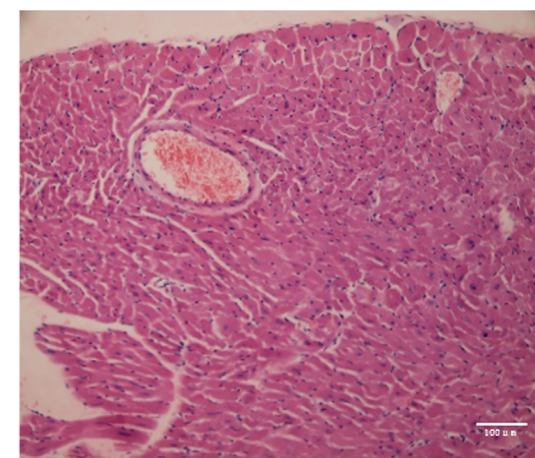

Sham-12w

Supplement: Supplementary file 1 — Fig S1 [file JCMM-26-1169-s007.pdf]
